# Improving outcomes after autologous transplantation in relapsed/refractory Hodgkin lymphoma: a European expert perspective

**DOI:** 10.1186/s12885-020-07561-2

**Published:** 2020-11-10

**Authors:** Anna Sureda, Marc André, Peter Borchmann, Maria G. da Silva, Christian Gisselbrecht, Theodoros P. Vassilakopoulos, Pier Luigi Zinzani, Jan Walewski

**Affiliations:** 1 Hematology Department, Hematopoietic Stem Cell Transplant Programme, Institut Català d’Oncologia-Hospital Duran i Reynals, Gran Via de l’Hospitalet, 199 – 203, 08908 Barcelona, Spain; 2 Institut d’Investigació Biomèdica de Bellvitge (IDIBELL), Universitat de Barcelona (UB), Barcelona, Spain; 3 Department of Hematology, Université catholique de Louvain, CHU UCL Namur, Yvoir, Belgium; 4 Department of Internal Medicine I, University Hospital Cologne, Cologne, Germany; 5 Department of Hematology, Instituto Português de Oncologia - Francisco Gentil, Lisbon, Portugal; 6 Institut d’Hématologie, Hôpital Saint Louis, Paris, France; 7 Department of Haematology and Bone Marrow Transplantation, National and Kapodistrian University of Athens, Laikon General Hospital, Athens, Greece; 8 Azienda Ospedaliero-Universitaria di Bologna, Bologna, Italy; 9 Istituto di Ematologia “Seràgnoli”, Dipartimento di Medicina Specialistica, Diagnostica e Sperimentale Università degli Studi, Bologna, Italy; 10 Department of Lymphoid Malignancies, Maria Sklodowska-Curie Institute Oncology Center, Warszawa, Poland

**Keywords:** Consolidation, HL, Salvage, Stem cell transplantation

## Abstract

**Abstract:**

Autologous stem cell transplantation (ASCT) is a well-established approach to treatment of patients with relapsed/refractory (R/R) Hodgkin lymphoma (HL) recommended by both the European Society for Medical Oncology and the National Comprehensive Cancer Network based on the results from randomized controlled studies. However, a considerable number of patients who receive ASCT will progress/relapse and display suboptimal post-transplant outcomes. Over recent years, a number of different strategies have been assessed to improve post-ASCT outcomes and augment HL cure rates. These include use of pre- and post-ASCT salvage therapies and post-ASCT consolidative therapy, with the greatest benefits demonstrated by targeted therapies, such as brentuximab vedotin. However, adoption of these new approaches has been inconsistent across different centers and regions.

In this article, we provide a European perspective on the available treatment options and likely future developments in the salvage and consolidation settings, with the aim to improve management of patients with HL who have a high risk of post-ASCT failure.

**Conclusions:**

We conclude that early intervention with post-ASCT consolidation improves outcomes in patients with R/R HL who require ASCT. Future approvals of targeted agents are expected to further improve outcomes and provide additional treatment options in the coming age of personalized medicine.

## Background

Hodgkin lymphoma (HL) is considered to be a curable hematological malignancy. The currently available chemotherapies and targeted therapies deliver frontline cure rates of approximately 90% for patients with early- or intermediate-stage disease and 70–90% for patients with advanced-stage disease [1–7]. For patients who either relapse or are refractory to frontline treatment, the standard of care comprises salvage chemotherapy followed by autologous stem cell transplantation (ASCT) [8–11]. Despite this intensified approach, up to 50% of patients will experience progressive disease following ASCT [9, 12, 13], representing a population with high-risk disease. This led to initiation of clinical studies in the 1990s to improve post-ASCT outcomes and consequently improve HL cure rates [14, 15], many of which showed less than satisfactory results, until the introduction of post-ASCT consolidation with brentuximab vedotin, an anti-cluster of differentiation 30 (CD30) antibody-drug conjugate (ADC). Brentuximab vedotin is able to improve progression-free survival (PFS) in patients with relapsed/refractory (R/R) classical HL (cHL) and a high risk of relapse post-ASCT [16, 17], and following its regulatory approval in the R/R cHL consolidation setting, a new generation of targeted therapies are being developed for use in this high-risk population.

This article will provide a European perspective on the optimal treatment of patients with high-risk R/R HL in the peri-ASCT setting, based on the current data from clinical studies and real-world evidence, with a focus on use of targeted agents to improve post-ASCT outcomes.

### Historical perspective on the role of ASCT in R/R HL

Two randomized studies provide the basis for the use of high-dose chemotherapy (HDC) plus ASCT as a standard of care in patients with R/R HL. The British National Lymphoma Investigation compared the activity of a combination of carmustine, etoposide, cytarabine, and melphalan (BEAM) plus autologous bone marrow transplant (ABMT) versus up to 3 cycles of mini-BEAM (lower doses of the BEAM drug combination) without ABMT in patients with R/R HL [18]. Although the study failed to show a significant difference in overall survival (OS) between arms, there were significant differences in favor of BEAM + ABMT in event-free survival (EFS; *p* = 0.025) and PFS (*p* = 0.005).

The BEAM-ASCT regimen was also assessed by the German Hodgkin Study Group (GHSG) and the Lymphoma Working Party of the European Group for Blood and Marrow Transplantation (EBMT) versus dexamethasone plus BEAM (Dexa-BEAM) [9]. After 3 years’ follow-up, there was no significant difference in OS between treatment groups; however, freedom from treatment failure (FFTF) was significantly improved in the BEAM-ASCT group compared with the Dexa-BEAM group (55% vs 34%; *p* = 0.019).

Despite the non-significant survival benefit with BEAM + ABMT/ASCT, this strategy was established as the historical standard of care based on the clear benefits on tumor control over conventional-dose salvage therapy alone. The lack of a demonstrable OS benefit of HDC + ASCT when compared with conventional chemotherapy in these clinical studies may be attributed to the small number of patients in the studies. A later detailed meta-analysis of data from the two studies suggested a trend towards an OS advantage for patients receiving HDC + ASCT compared with conventional chemotherapy alone (hazard ratio [HR] 0.67, *p* = 0.10) [19]. In addition, the use of ASCT later in the course of the disease may have confounded detection of any potential effects of HDC + ASCT on OS.

### Improving post-ASCT outcomes in HL: pre-transplant strategies

#### Current conventional salvage chemotherapy

The most widely investigated strategy to improve post-ASCT outcomes is the optimization of pre-ASCT salvage chemotherapy. A variety of regimens have been studied, including platinum-based dexamethasone, cytarabine, and cisplatin/doxorubicin, methylprednisolone, high-dose cytarabine, and cisplatin/etoposide, methylprednisolone, high-dose cytarabine, and cisplatin (DHAP/ASHAP/ESHAP [20–24]), ifosfamide-based (MINE/ICE/IVE/IVOx [25–29]), and gemcitabine-based combinations (GVD/IGEV/GDP/GemOx/BeGEV) [30–34]. Most of these studies evaluated the efficacy of salvage chemotherapy in terms of objective responses and the impact on OS and post-transplant PFS; reports of the impact on stem cell mobilization, stem cell quality, and stem cell transplantation (SCT) rates are inconsistent (Table 1). Objective response rates (ORRs) ranged from 61% with GVD (in ASCT-naïve patients) to 88/89% with ICE and DHAP [20, 26, 27], and complete response (CR) rate was as low as 17% with GDP and as high as 73% with BeGEV [32, 34]. PFS was 78% with ESHAP in patients who achieved CR, but 16% in those who achieved partial remission (PR) [24]; ICE treatment resulted in a PFS of 70% [35], but only 53 and 62% with IGEV and BeGEV, respectively [31, 34]. EFS was 36% with ASHAP and 68% with ICE [22, 26]. OS with ESHAP was estimated at 35% at 3 years in one study and 73% at 5 years in another study [23, 24]. However, the absence of prospective randomized studies comparing the regimens makes it impossible to reach conclusions regarding the superiority of particular combinations. This is reflected in the current European Society for Medical Oncology (ESMO) and National Comprehensive Cancer Network (NCCN) treatment guidelines, which do not recommend any specific salvage therapy regimen for patients with R/R HL [10, 11, 36]. Instead, appropriate therapy is generally selected based on patient-related factors, familiarity of the treatment center with particular regimens, treatment as in-patients versus out-patients, and the toxicity profile of each salvage regimen – patients with concomitant coronary, pulmonary, or renal diseases may require different salvage management.

**Table 1:** Conventional chemotherapy-based salvage regimens assessed in patients with R/R HL

Regimen(s)	Patients, ***N***	Pre-ASCT response rate	Survival data in overall patient group (± ASCT)	
MINE [25]	100	ORR = 75%	2-year survival rate	59%
ASHAP [22]	56	ORR = 70% (CR = 34%, PR = 36%)	OS, 5-year follow-up	41%
			EFS, 5-year follow-up	36%
DHAP [20]	102	ORR = 88%, (CR = 21%, PR = 67%)	NR	NR
ESHAP [23]	22	ORR = 73%	50-month follow-up	32% alive and disease-free
			3-year estimates	OS = 35% Disease-free = 27%
ESHAP [24]	82	ORR = 67% (CR = 50%)	OS, 5-year follow-up	72.6%
			PFS, 5-year follow-up	• Pts achieving CR = 78% • Pts achieving PR = 16% *p* < 0.01)
ICE [26]	65	ORR = 88% (CR = 26%, PR = 58%)	OS, 43-month follow-up	83%
			EFS, 43-month follow-up	68%
ICE [27]	R/R HL (*n* = 13) non-HL (*n* = 62)	ORR = 89% (CR = 29%, PR = 60%)	OS, 24-month follow-up	65% (all pts)
			EFS, 24-month follow-up	42% (all pts)
ICE/aICE [35]	97	NR	OS, 51-month follow-up	80%
			PFS, 51-month follow-up	70%
IVE [28]	51	ORR = 84% (CR = 60%, good PR = 8%, PR = 16%)	NR	NR
IVOx [29]	34	ORR = 76% (CR = 32%)	OS, 5-year follow-up	74%
			EFS, 5-year follow-up	63%
GVD pre- or post-ASCT [30]	94	ASCT-naïve pts. ORR = 61% Prior-SCT pts. ORR = 75%	OS, 3.6-year follow-up	• SCT-naïve pts. median ORR = 61% • Prior-SCT pts. ORR = 75%
			EFS, 3.6-year follow-up	• SCT-naïve pts. median EFS not reached, 52% progression-free at 4 years • Prior-SCT pts. median EFS duration 8.5 months
IGEV [31]	91	CR = 53.8%, PR = 27.5%	OS, 3-year follow-up	70.03%
			PFS, 3-year follow-up	52.98%
BeGEV [34]	59	ORR = 83% (CR = 73%, PR = 10%)	OS, 2-year follow-up	77.6%
			PFS, 2-year follow-up	62.2%
GDP [32]	23	ORR = 69.5% (CR = 17.3%, PR = 52.2%)	NR	NR
GemOx [33]	24	ORR = 71% (CR = 38%, PR = 33%)	OS, 3-year follow-up	Median of 26 months
			PFS, 3-year follow-up	Median of 14 months

*NR* not reported, *Pts* patients

Patients who achieve a response, particularly a CR, with conventional salvage chemotherapy prior to ASCT, are likely to have an improved clinical outcome compared with patients who have partially or totally chemo-resistant disease at relapse. Moskowitz et al. reported an analysis of long-term outcomes in 75 patients demonstrating significant improvements (*p* < 0.001) in EFS (60% vs 19%), PFS (62% vs 23%), and OS (66% vs 17%) in patients who responded to standard-dose second-line therapy after relapse, compared with those who had a poor response, respectively [37]. Similarly, in a prospective analysis of 195 patients treated over a 20-year period, patients who had achieved a CR with conventional salvage chemotherapy and who were still in CR at the time of ASCT had a 5-year OS of 79%, which dropped to 59% for those in PR, and 17% for those with resistant disease (*p* < 0.0001). Corresponding 5-year PFS rates were 69% versus 44% versus 14% (*p* < 0.0001) [38]. More recently, in a phase II study of 97 patients, those who proceeded to SCT with a positron-emission tomography (PET)-negative status achieved an EFS of > 80%, compared with 29% in PET-positive patients [35]. Collectively, these studies demonstrate that the aim of any modern salvage therapy is to produce a deep remission and PET-negative status prior to undergoing HDC-ASCT. However, ASCT should not be solely withheld due to lack of PET-negativity [39].

#### Can salvage therapy be further improved with conventional cytotoxic agents?

The GHSG have investigated the concept of escalated pre-ASCT chemotherapy, delivered as sequential-HDC, compared with standard HDC, in an effort to improve treatment outcomes for patients with R/R HL receiving HDC + ASCT [40]. In the GHSG study of standard versus intensified BEAM-ASCT following DHAP in patients with relapsed HL (*n* = 241), there were no significant differences in FFTF (*p =* 0.56) or OS (0.82) between the study arms. Toxicity was considerably higher in the intensified arm, with increased rates of grade 3/4 adverse events (AEs), although this did not translate into increased mortality.

Patients who achieve a CR following induction chemotherapy are highly likely to respond to post-relapse interventions [31]. Previous studies in patients with HL who have experienced multiple relapses have demonstrated that bendamustine monotherapy has promising activity, with patients who were ineligible for ASCT, or for whom ASCT had failed, achieving CR rates of 25–35% [41–43]. In an open-label phase II study, ORR was 83% (CR = 73%) following 4 cycles of BeGEV as induction therapy before ASCT in 43/49 patients. Two-year PFS and OS rates in the overall patient population were 62.2 and 77.6%, respectively, and 80.8 and 89.3% among patients who underwent ASCT [34].

#### Optimization of salvage therapy with the use of brentuximab vedotin

Brentuximab vedotin is currently approved as a monotherapy for adult patients with R/R CD30-positive cHL following ASCT or following ≥ 2 prior therapies when ASCT or multi-agent chemotherapy is not a treatment option [44]. The pivotal phase II SG035–0003 study of brentuximab vedotin (1.8 mg/kg every 3 weeks [Q3W]) after failed ASCT in 102 patients with R/R HL, reported median OS of 22.4 months and median PFS of 5.6 months at the primary analysis [45]. With prolonged follow-up (median 35.1 months) estimated 5-year OS and PFS rates were 41 and 22%, respectively [46]. Corresponding OS and PFS rates amongst the 33% of patients who achieved a CR were 64 and 52%, and the median response duration was not reached. The prospective phase IV C25007 study evaluated brentuximab vedotin (1.8 mg/kg Q3W) in 60 R/R HL patients who were unsuitable for ASCT or multi-agent chemotherapy [47]. The ORR was 50% (CR = 12%), 47% of patients were bridged to ASCT, and the estimated 12-month OS was 86%, thus enabling patients with high-risk disease to receive ASCT, even if they had a suboptimal response to frontline treatment or chemotherapy/radiotherapy-based salvage.

Lately, brentuximab vedotin has been assessed alone and in combination with conventional chemotherapy-based regimens for salvage therapy prior to ASCT in several studies (Table 2). A phase II study assessed 4 cycles of standard-dose, single-agent brentuximab vedotin (1.8 mg/kg Q3W) as salvage treatment following induction therapy with doxorubicin, bleomycin, vinblastine, and dacarbazine (ABVD) and/or bleomycin, etoposide, doxorubicin, cyclophosphamide, vincristine, procarbazine, and prednisone (BEACOPP) [52]. In 37 patients, ORR was 69% and CR rate was 35%; 89% of patients were able to proceed to ASCT, either with or without additional chemotherapy.

**Table 2:** Brentuximab vedotin-based salvage regimens assessed in patients with R/R HL

Regimen(s)	Patients	Response before transplant	OS	PFS
B + DHAP [48]	R/R HL (*N* = 61)	• Metabolic CR 79% • Metabolic PR 8% • Progressive disease 7% • 87% of pts. were mobilized and received ASCT	2-year OS 92%	2-year PFS 76%
BrESHAP [49]	R/R HL after frontline chemotherapy (*N* = 66)	• ORR 91% • CR 82% • PR 10% • 64 pts. were mobilized and 60 received SCT	30-month OS 91%	30-month PFS 71%
PET-adapted brentuximab vedotin + aICE [16]	R/R HL who had failed one previous doxorubicin regimen (*N* = 46)	• ~ 30% of pts. achieved PET-negativity with brentuximab vedotin alone • aICE increased PET-negativity rates to ~ 80%	NR	2-year EFS = 82%
PET-adapted brentuximab vedotin + aICE [50, 51]	First relapse or primary refractory CD30+ cHL (*N =* 24 [50]) (*N* = 42 [51])	• 87% CR per investigator, 70% per independent review [50] • 69.2% CMR [51]	NR	1-year PFS estimate 69% (95% CI 53–81%)
Brentuximab vedotin [52]	R/R HL (*N* = 37)	• Best ORR = 69% (CR = 33%) • 12 pts. with CR received SCT • 11/13 pts. with PR and all pts. with SD/PR required additional chemotherapy	NR	NR
Brentuximab vedotin + bendamustine [53]	R/R HL (*N* = 55)	• ORR 92.5% • CR 73.6% • 41 patients were mobilized and 40 underwent ASCT	NR	2-year PFS 62.6%
Brentuximab vedotin + nivolumab [54]	R/R HL (*N* = 61)	• ORR 82% • CR 61% • 54 pts. underwent ASCT	NR	6-month estimated PFS 89%

Encouraging results with PET-adapted salvage therapy have been reported by Moskowitz et al. [16] in a phase II open-label study to assess the efficacy of 2 cycles of brentuximab vedotin-based salvage (1.2 mg/kg on days 1, 8, and 15) in patients with R/R HL who had failed one previous doxorubicin-containing regimen. Following the first 2 cycles of brentuximab vedotin, 27% of patients were PET-negative and proceeded directly to ASCT and 69% initiated augmented ifosfamide, carboplatin, and etoposide (aICE) [16]. Seventy-six percent of patients achieved PET-negativity prior to HDC/ASCT, thus maximizing the potential for improved post-ASCT outcomes. Patients treated with 3 cycles of brentuximab vedotin also achieved similar rates of PET-negativity (30%) and proceeded directly to ASCT, whilst the remainder received either aICE or ICE [39]. Prior to transplant, 80% of patients had achieved PET-negativity with either brentuximab vedotin alone or combined with aICE/ICE, and with 2 years’ follow-up EFS was 82%.

Brentuximab vedotin combined with standard ICE has also shown promise in phase I/II clinical studies [50, 51]. In the NCT02227199 study, 20/23 evaluable patients previously treated with ABVD achieved a PET CR per investigator review following brentuximab vedotin + ICE; 70% were in PET CR per independent central review [50]. Final data from the Lymphoma Academic Research Organisation phase I/II study showed that 69% (*n =* 27/39) of patients who received brentuximab vedotin (1.8 mg/kg) plus ICE achieved a complete metabolic response (CMR) and 26% achieved a partial metabolic response. Twenty patients in CMR went on to receive ASCT [51]. Neither study reported any unexpected toxicities.

Two brentuximab vedotin + platinum-based chemotherapy combinations appear particularly promising: brentuximab vedotin + DHAP (B + DHAP), and brentuximab vedotin + ESHAP (BrESHAP). In the phase II BRaVE study, the high metabolic CR rate of 79% achieved with B + DHAP suggests that this approach is worth investigating further [48]. Moreover, the Grupo Español de Linfomas y Trasplantes de Médula Ósea (GELTAMO) study further demonstrated the potential for brentuximab vedotin combinations with chemotherapy in the pre-ASCT setting. After approximately 27 months of follow-up, 74% of patients were FFTF, PFS was 71%, and OS was 91% [49].

The combination of brentuximab vedotin (1.8 mg/kg Q3W) and bendamustine (90 mg/m^2^ days 1–2 Q3W) was highly active as first salvage therapy for patients with R/R HL in a phase I/II study involving 55 patients [53]. ORR was 93% and CR was 74% after a median of 2 cycles of therapy.

Interim results of another phase I/II study have also demonstrated the utility of 4 cycles of a brentuximab vedotin (1.8 mg/kg Q3W) combined with nivolumab (3.0 mg/kg Q3W) combination as initial salvage therapy for patients with R/R HL [54]. ORR in all treated patients (*n =* 61) was 82%, and CR was 61%.

In summary, single-agent brentuximab vedotin as a salvage therapy shows activity that is not that different to that seen with classical chemotherapy regimens, albeit with a lower systemic toxicity, although these have not been compared in a prospective, randomized phase III study. Clinical studies of brentuximab vedotin combined with chemotherapy build on the demonstrated efficacy of brentuximab vedotin monotherapy, with high CR rates observed with combination therapy, and with no new safety signals. Again, these observations need to be confirmed in prospective, randomized phase III studies, such as the BRESELIBET study (NCT04378647) that was opened to enrolment in June 2020. Despite the low patient numbers in these studies, brentuximab vedotin plus chemotherapy regimens are frequently used as salvage therapy in the clinic.

### Tandem SCT as a strategy to improve ASCT outcomes

The successful implementation of aggressive tandem transplant programs in myeloma led the Lymphoma Study Association (LYSA) and the Société Francaise de Greffe de Moelle (SFGM) to conduct an exploratory joint phase II study to assess the feasibility of tandem ASCT in patients with high-risk HL, defined as primary refractory disease or ≥ 2 of the following risk factors at first relapse: time to relapse < 12 months, stage III/IV at relapse, and relapse within previously irradiated sites [55]. In the high-risk group, 5-year freedom from second failure rate was 46%, which was higher than the 30% reported by historical studies, and OS rate was 57%. After a median follow-up of 10.3 years, respective failure-free survival and OS rates were 64 and 70% for intermediate-risk patients and 41 and 47% for high-risk patients [56].

In 2018, the Southwest Oncology Group published results from a phase II study to assess the efficacy of tandem ASCT for 98 patients with primary progressive or recurrent HL [57]. After a median of 6.2 years’ follow-up, 2-year and 5-year PFS rates were 63 and 55%, and 2-year and 5-year OS rates were 91 and 84%, respectively.

Tandem SCT utilizing ASCT followed by an allogeneic SCT (allo-SCT) was assessed by the LYSA in a multicenter observational study of 120 patients with high-risk R/R HL who were prospectively registered on a French national database [58]. After 43 months’ median follow-up the 2-year PFS and OS rates were 71 and 85%, respectively, in tandem-transplanted patients, but with no significant difference between ASCT–allo-SCT or tandem ASCT options.

A retrospective analysis of patients with R/R HL who received tandem SCT between January 2004 and December 2015, performed by the Lymphoma Working Party of the EBMT, found that dual SCT might be effective in high-risk populations [59]. Three years after dual SCT, PFS was 53%, OS was 72, 34% of patients had relapsed, and 13% had non-relapse mortality.

These clinical studies suggest that there may be a place for tandem SCT in the treatment of R/R HL, although the exact patient subgroup that will derive the most benefit from this strategy remains to be defined. These strategies may become less relevant in the era of novel agents. Integration of pre-ASCT PET-response assessment, post-ASCT salvage and consolidation therapies, including targeted agents, may offer higher survival rates with less intensive regimens.

### Improving post-ASCT outcomes in HL: post-transplant strategies

Despite efforts to optimize pre-ASCT strategies, many patients will still relapse after receiving ASCT [60]. Use of post-ASCT consolidation, even when there is no detectable residual lymphoma, can prevent or delay relapse or progression. Prospective and retrospective studies have demonstrated improved PFS in patients who received post-ASCT rituximab maintenance compared with no maintenance in patients with follicular lymphoma [61, 62]. These studies suggest that maintenance therapy plays a role in suppressing emergence of minimal residual disease (MRD) leading to relapse. A randomized study by Le Gouill et al., demonstrated clearance of MRD and improved PFS and OS in patients with mantle cell lymphoma (MCL) treated with post-ASCT rituximab consolidation for 3 years [63]. The current HL treatment guidelines fail to emphasize the importance of the timing of such medical intervention. As relapse/progression tends to occur soon after ASCT, the greatest therapeutic effect is likely to be achieved with a consolidation treatment delivered as early as possible after receipt of ASCT [64].

To ensure that patients are exposed to an effective consolidation regimen for as long as possible, it is important that consolidative therapies deliver a combination of efficacy and acceptable tolerability for patients. Novel targeted therapies that are either approved or are under investigation in this setting are discussed below and summarized in Table 3.

**Table 3:** Novel targeted post-ASCT consolidative options in development or approved for use in R/R HL

Targeted agent	Treatment setting	Efficacy	Safety
**ADCs**			
Brentuximab vedotin (anti-CD30; monotherapy) phase III AETHERA study (NCT01100502) [17, 65]	Consolidation therapy after ASCT in patients with HL at high risk of progression or relapse (*n =* 165 brentuximab vedotin arm)	• 2-year PFS rate: 63% • 5-year PFS rate: 59%	• Grade 3/4 AEs: 16% • Peripheral neuropathy: 67% (90% of which had improved or completely resolved after 5 years’ follow-up)
Camidanlumab tesirine (anti-CD25; monotherapy) phase I study (NCT00516217) [66]	R/R HL (median 5 prior lines; *n* = 26)	• ORR 81% • CR 50%	• Grade ≥ 3 treatment-emergent adverse events: 62%
**Histone deacetylase inhibitors**			
Panobinostat (monotherapy) phase III (NCT01034163) [67]	Consolidation therapy after HDC and ASCT in patients with HL at high risk of relapse (*n* = 27 panobinostat arm)	Study discontinued early due to poor accrual (41/367 planned patients enrolled), so efficacy not formally evaluated	• Grade 3/4 AEs: 65%. Most frequent: - Neutropenia (27%) - Thrombocytopenia (15%) - Diarrhea, vomiting, fatigue (12%)
**Monoclonal antibodies**			
Nivolumab (anti-PD-1; monotherapy) phase II CheckMate 205 study (NCT02181738) [68, 69]	R/R cHL after ASCT and brentuximab vedotin	• ORR 69%	• Grade 3/4 AEs were low
Pembrolizumab (anti-PD-1; monotherapy) (NCT02362997) [70]	Post-ASCT consolidation therapy in patients with R/R cHL who had achieved a CR or PR with salvage chemotherapy (*N* = 31)	• PFS rate at 18 months: 82% • OS rate at 18 months: 100%	• Grade ≥ 2 AEs: 80% • Grade ≥ 3 AEs: 30% • Immune-related AEs: 43%
Galiximab (anti-CD80) phase II CALGB 50602 study (NCT00516217) [71]	R/R HL; median 3 prior regimens (*n* = 29)	• ORR 10.3% • Median PFS 1.6 months	• Minimal grade 3/4 toxicities
Lucatumumab (anti-CD40) phase Ia/II study (NCT00670592) [72]	R/R HL (*n* = 37)	• ORR 13.5%	• Grade 3/4 AEs: 65% (HL and NHL)
TNX-650 (anti-IL-13) phase I/II study (NCT00441818)	Refractory HL	• Study in progress	
Relatlimib (anti-LAG-3) phase I/II study with/without nivolumab (NCT02061761)	R/R HL	• Study in progress	
AMG655 (anti-TRAIL) with bortezomib or vorinostat phase Ib study (NCT00791011)	R/R Lymphoma	• No longer in development	
RFT5-SPMT-dgA (anti-CD25)	R/R HL	• No longer in development	
Alemtuzumab (anti-CD52) phase II study with dose-adjusted-EPOCH regimen (NCT01030900)	R/R HL	• Study in progress (monotherapy failed)	
**Bispecific antibody**			
AFM13 (anti-CD30/CD16a; monotherapy) phase I study complete; phase II ongoing (NCT02321592) [73]	Heavily-pretreated R/R HL (*n* = 28)	• ORR 11.5%	• Grade ≥ 3 AEs: 29%
AFM13 (anti-CD30/CD16a; plus pembrolizumab) phase Ib KEYNOTE-206 study (NCT02665650) [74]	R/R HL (failed brentuximab vedotin; *n* = 30)	• ORR 88% • CR 46% • 6-month PFS 77%	• Grade 3/4 AEs included infusion-related reactions in 13%
**CAR-T cells**			
Anti-CD30 RELY-30 phase I study (NCT02917083) [75]	R/R HL Median 5 prior therapies (*n* = 14)	• CR 58%	• Not reported
Phase Ib/II study [76]	R/R HL Median 7.5 prior therapies (*n* = 22)	• CR 53%	• Not reported
Phase I study (NCT02259556) [77]	R/R HL Heavily pretreated patients (*n* = 18)	• ORR 39% • Median PFS 6 months	• Grade ≥ 3 AEs in 2 patients
Anti-LMP1/2 phase I study (NCT00671164) [78]	R/R HL/NHL (*n* = 25)	• 2-year EFS 50% • ORR 62% • CR 52%	• Not reported

*EPOCH etoposide, prednisone, vincristine, cyclophosphamide, and doxorubicin; NHL* non Hodgkin lymphoma

#### Brentuximab vedotin as consolidation therapy

Consolidation therapy is administered to patients who may have undetectable residual lymphoma and those who may already be cured. Therefore, the optimal consolidation therapy should have a favorable/low toxicity profile that does not impair hematopoietic and immunological recovery post-ASCT, with minimal impact on non-lymphoid/hematopoietic tissues. As biologic treatment options, such as brentuximab vedotin, are less toxic than traditional cytotoxic chemotherapy, they may be an ideal treatment option in this setting.

The utility of brentuximab vedotin as early consolidation therapy after ASCT in patients with high-risk HL was explored in the phase III AETHERA study (see Table 3) [65]. Patients enrolled in AETHERA (*n =* 329) had one of the following pre-ASCT risk factors for relapse: primary refractory HL (i.e., CR not achieved with frontline chemotherapy), relapsed HL with a duration of initial remission < 12 months, or extranodal involvement at the start of pre-ASCT HDC. After a median of 30 months’ follow-up, PFS per independent review was significantly longer with brentuximab vedotin compared with placebo (median PFS of 42.9 vs 24.1 months [HR 0.57; 95% CI 0.40–0.81]; *p =* 0.0013) [65]. PFS benefit was consistent across pre-specified subgroups, including patients with primary refractory HL and those who relapsed < 12 months after frontline therapy. After 5 years of follow-up, brentuximab vedotin continued to demonstrate sustained PFS benefit (HR 0.52; 95% CI 0.38–0.72), with 5-year per investigator PFS rates of 59% versus 41% with placebo, implying that a considerable proportion of these patients might have been cured [17]. This long-term PFS benefit with brentuximab vedotin was more pronounced in patients with higher numbers of pre-ASCT risk factors, which included relapse within < 12 months or refractoriness to frontline therapy, best response of PR or stable disease (SD) to most recent salvage therapy, extranodal disease at pre-ASCT relapse, B-symptoms at pre-transplant relapse, or ≥ 2 prior salvage therapies. An OS analysis has not yet been performed, as the planned number of events has not been reached. Brentuximab vedotin consolidation was well tolerated in the AETHERA study, with 93 of 167 patients (56%) experiencing grade 3/4 AEs [65]. Sixty-seven percent of brentuximab vedotin-treated patients experienced some level of peripheral neuropathy, 90% of which had either completely resolved or decreased in severity after 5 years’ follow-up [17].

The results of the AETHERA study are reflected in the HL treatment guidelines developed by ESMO and NCCN, and the lymphoma post-ASCT maintenance therapy guidelines developed by a joint expert panel consisting members of American Society for Blood and Marrow Transplantation (ASBMT), Center for International Blood and Marrow Transplant Research (CIBMTR), and EBMT [10, 11, 36]. ESMO guidelines recommend consolidation with brentuximab vedotin following HDC-ASCT in patients presenting with ≥ 1 of the following risk factors: primary disease progression, early disease recurrence after the end of frontline treatment (< 12 months), and extranodal disease at the time of relapse, without specifying the duration of consolidation therapy [11]. NCCN recommend brentuximab vedotin for 1 year for patients with a high risk of relapse, defined as ≥ 2 of the following risk factors: remission lasting < 1 year, extranodal involvement, PET-positive response at time of transplant, B symptoms, and/or > 1 salvage/subsequent therapy regimen [10]. The ASBMT, CIBMTR, and EBMT panels recommend post-ASCT consolidation with brentuximab vedotin for a maximum of 16 cycles every 3 weeks, or until unacceptable toxicity or disease relapse (whichever occurs first) for brentuximab vedotin-naïve patients with cHL and one or more high-risk features as defined by the AETHERA study [36]. The panel do not recommend brentuximab vedotin post-ASCT consolidation in patients whose HL has shown signs of refractoriness to prior treatment with brentuximab vedotin; however, high-risk patients with relapsed disease and limited prior exposure to brentuximab vedotin (~ 4–6 cycles) prior to ASCT and no evidence of brentuximab-vedotin-refractory disease, were recommended for brentuximab vedotin consolidation.

#### Novel consolidation options

Following demonstration of the efficacy of brentuximab vedotin as consolidation in post-ASCT HL treatment, clinical studies to assess the efficacy of other anti-tumor agents, such as chemotherapies and checkpoint inhibitors, have been initiated. Novel therapies including ADCs, bispecific antibodies, and chimeric antigen receptor-modified T (CAR-T) cells are under investigation in R/R HL and other lymphomas, and may be an option for post-ASCT consolidation in the future. These are discussed briefly below.

##### ADCs

One other ADC is in development for the treatment of R/R HL. Camidanlumab tesirine is a human anti-CD25 monoclonal antibody conjugated to a potent pyrrolobenzodiazepine dimer toxin. A phase I, first-in-human study reported an ORR of 81% in 26 patients with R/R cHL in the 45 μg/kg dose group, with 50% CR. Grade ≥ 3 treatment-emergent adverse events were reported in 62% of patients (Table 3) [66].

##### Histone deacetylase inhibitors

Panobinostat has been investigated as consolidation therapy for patients with HL in a phase III study that was discontinued due to poor accrual [67].

##### Nivolumab

Preclinical analyses of lymphoma cells revealed the presence gene amplification of programmed cell death-1 (PD-1) ligand [79], raising the prospect of anti-PD-1 agents as therapeutic agents in HL. Nivolumab, a fully human anti-PD-1 monoclonal antibody, was approved for use as monotherapy in adults with R/R cHL after ASCT and brentuximab vedotin based on the results of the phase II CheckMate 205 study [68, 69]. After a median follow-up of 18 months, ORR per independent review was 69%, ranging from 65 to 73% across brentuximab vedotin-naïve and pre- and post-ASCT brentuximab vedotin-treated cohorts (Table 3). The safety and efficacy of nivolumab consolidation is being assessed in patients with HL, who are at risk of relapse or progression after receipt of an ASCT, in a single-arm phase II study (NCT03436862).

##### Pembrolizumab

Pembrolizumab is a humanized anti-PD-1 monoclonal antibody that is also being investigated in the relapsed HL post-ASCT consolidation setting [80]. In the phase II KEYNOTE-087 study of pembrolizumab in patients with R/R cHL, ORR per independent review was 72% (95% CI 65–78) after a median follow-up of 27.6 months [81]. The activity of pembrolizumab is also being assessed by Armand et al. in an ongoing multi-cohort phase II study in patients with R/R cHL who had achieved a CR or PR with first salvage chemotherapy (see Table 3) [70]; in contrast in the AETHERA study, patients were required to have achieved a CR, PR, or SD with salvage chemotherapy [65]. The primary endpoint, PFS at 18 months, was met, with a PFS rate of 82% for the evaluable patients; OS rate at 18 months was 100%.

##### Other monoclonal antibodies

In addition to PD-1, other targets under investigation include CD80, CD40, IL-13, LAG-3, TRAIL, CD25, and CD52. Early data for the anti-CD80 monoclonal antibody galiximab, and lucatumumab, which targets CD40, are not very promising, with ORRs around 10–14% (Table 3) [71, 72]. Antibodies against CD25 and TRAIL are no longer under development [82].

##### Bispecific antibodies

The bispecific antibody AFM13 targets CD30 and CD16a to recruit natural killer cells to CD30-positive malignancies. A monotherapy study in 26 heavily-treated R/R HL patients demonstrated mild efficacy (ORR 11.5%, rising to 23% in the higher dose group; *n* = 13) with a good safety profile [73]. A phase Ib study (KEYNOTE-206) combining AFM13 with pembrolizumab resulted in a much improved ORR of 88% (Table 3), which also compares favorably with rates seen with pembrolizumab alone [74].

##### CAR-T cells

CAR-T cell therapies targeting CD30, CD123, and Epstein-Barr virus (EBV)-related proteins are in development for the treatment of cHL, and are showing promising early results. Two US-based groups and one group in China have investigated CD30 CAR-T cell therapy in heavily-pretreated R/R HL with ORR rates of 67, 63, and 39%, respectively (Table 3) [75–77]. A pivotal phase II study (NCT04268706) is being planned based on these encouraging results, and the US Food and Drug Administration granted Regenerative Medicine Advanced Therapy designation to this treatment on March 2, 2020. CAR-T cell therapy targeting the EBV antigens latent membrane protein 1 (LMP1) and LMP2 has also shown an encouraging ORR of 62%, with 52% CR, in patients with R/R lymphoma [78].

##### Post-ASCT radiotherapy

HL is extremely sensitive to radiotherapy, even after multiple lines of chemotherapy; however, there is a lack of large prospective randomized studies to evaluate the role of post-ASCT consolidation radiotherapy. It is generally believed that radiation-naïve patients with localized residual lymphoma post-salvage therapy or post-ASCT, could benefit from radiotherapy before signs of progressive systemic lymphoma develop. Retrospective analyses of patients treated with radiotherapy as a post-ASCT salvage or consolidation option in the clinic have shown variable effectiveness.

In the post-ASCT consolidation setting, radiotherapy has shown some promise with significant improvements in local control at 36 months post-ASCT in patients receiving radiotherapy compared with no radiotherapy (78% vs 48%, *p =* 0.02) accompanied by limited toxicity [83]. Wilke et al. found more promising results in radiotherapy vs no radiotherapy regimens, with significant improvements in 2-year PFS (67% vs 42%, *p* < 0.01), which could also be seen in patients with bulky disease (62% vs 39%, *p =* 0.02), B-symptoms (48% vs 28%, *p =* 0.05), primary-refractory disease (47% vs 32%, *p =* 0.02), and those with a PR per pre-transplant imaging (47% vs 32%, *p =* 0.02) [84]. However, neither of these analyses were able to demonstrate a significant improvement in OS with consolidative radiotherapy.

A single-institution analysis of 56 patients by Goda et al. found that in patients who had failed ASCT and received radiotherapy as a salvage option (salvage radiation therapy [sRT] alone in 34 patients and sRT plus chemotherapy in 22 patients), ORR was 84% (CR = 36%; PR = 48%) with a median OS of 40.8 months and 5-year OS of 29% [85]. The 2-year systemic PFS of 16% and overall disease control rate of 17% were disappointing, whereas local PFS at 2 years was 65%, leading the authors to conclude that radiotherapy may be of use for palliation of incurable HL.

The paucity of consistent data supporting the use of post-ASCT radiotherapy are reflected in the heterogeneous adoption of this approach in European clinical practice, other than to treat localized disease.

#### The challenge of demonstrating OS benefit in post-ASCT R/R HL

Demonstration of an OS benefit remains the ideal clinical endpoint in the oncological studies, representing the time from randomization until death. Salvage with HDC-ASCT is an effective approach that can be further improved by consolidation strategies in high-risk patients [65]; however, owing to the effectiveness of targeted therapies applied later in the disease course, it can take many years to accumulate enough survival events to demonstrate a statistically significant difference in OS between an experimental therapy and the placebo/control arm. In the AETHERA study, patients had lengthy post-ASCT PFS and OS, with some patients not relapsing during follow-up, i.e., potentially achieving a cure [65]. After a median follow-up of 5 years there were still insufficient OS events to draw any conclusions on the efficacy of brentuximab vedotin compared with placebo in terms of impact on OS [17], and this may be further complicated by the use of subsequent checkpoint inhibitors. Crucially, AETHERA permitted patients in the placebo arm to crossover to the brentuximab arm, resulting in 87% of patients in the placebo arm eventually receiving brentuximab vedotin monotherapy as a subsequent therapy, further confounding assessment of the impact of brentuximab vedotin on OS [17, 65].

**Fig. 1:**
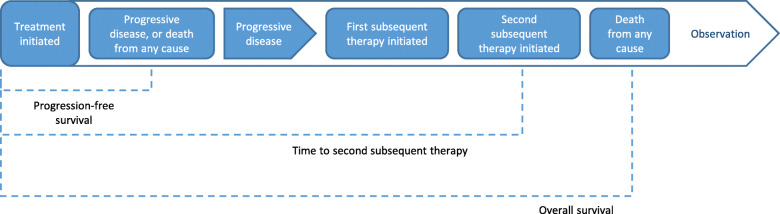
Time to second subsequent therapy as an endpoint. Progression-free survival is defined as the length of time between treatment initiation and progressive disease or death, whichever occurs first. Overall survival is defined as the length of time during and after the treatment of a disease until death from any cause. In contrast, time to second subsequent therapy is defined as the length of time between treatment initiation and the start of the third line of treatment (second subsequent therapy)

Based on the challenges faced when using OS as an endpoint, the European Medicines Agency recommended that time to second subsequent therapy (TTSST) is used as a measure of ongoing disease control and an alternative to OS (Fig. 1). TTSST is defined as the time between treatment initiation and the start of the third line of treatment (second subsequent therapy) and can be used to assess continuous lymphoma control in patient populations at high risk of relapse, in which multiple additional therapies are often used [86, 87].

In a post hoc analysis of the AETHERA study at 5 years, significantly fewer patients in the brentuximab vedotin arm had a TTSST event compared with the placebo arm (36% vs 46%, *p* < 0.0001), implying that 64% of brentuximab vedotin-treated patients had not received ≥ 2 subsequent therapies for HL or died, versus 54% in the placebo arm. Furthermore, subsequent transplants, including allo-SCT, were more common in the placebo arm than the brentuximab vedotin arm [17, 65].

## Discussion

The advent of treatment strategies and the consideration of CMR before ASCT has improved patient outcomes in the R/R HL setting; however, a considerable number of patients continue to progress or relapse and display dismal post-transplant outcomes. Appropriate utilization of post-ASCT consolidation now appears to be a promising and widely-applicable strategy.

The introduction of brentuximab vedotin as a post-ASCT consolidation option has significantly improved patient outcomes in terms of PFS (30% reduction in PFS events compared with placebo) and reduced the need for second subsequent therapy (36% of brentuximab vedotin patients compared with 46% of placebo patients) [17]. The persistence of these outcomes after 5 years’ follow-up demonstrate the benefits provided by brentuximab vedotin consolidation.

Currently, there is a paucity of published data and guidance on the utility of biomarkers in the R/R HL consolidation setting. The AETHERA study found that the benefit of brentuximab vedotin was more pronounced in patients with additional pre-ASCT risk factors for relapse. At 5 years, the HR for PFS was 0.42 (95% CI 0.30–0.60) in patients with ≥ 2 risk factors and 0.39 (95% CI 0.26–0.60) in those with ≥ 3 risk factors [17]. Therefore, prognostic factors should be considered when making treatment decisions in the R/R HL setting.

PET-positivity is a known negative prognostic factor; however, large-scale adequately-powered studies are required to obtain meaningful data to appropriately quantify the effect of the persistence and the magnitude of pre-ASCT PET-positivity on the outcome of ASCT. In the AETHERA study, PET scans before ASCT were not mandated in the study protocol, reflecting clinical practice at the time [65]. Although PET scans were performed in approximately two-thirds of patients, objective criteria were not required for interpretation. The benefit of brentuximab vedotin appeared to be reduced in patients who were PET-negative before ASCT, according to a univariate analysis. The role of biomarkers in this setting is evolving. PET scans are now a requirement for all studies and metabolic tumor volume is also emerging as a promising new prognostic biomarker.

MRD has been used as surrogate parameter for treatment effectiveness in follicular lymphoma and MCL [61, 62, 88–91] and further studies are needed to demonstrate the clinical utility of MRD as a biomarker for personalization of HL consolidation strategies. Despite the paucity of evidence supporting routine use of MRD assessment as part of consolidation, the marked PFS benefit seen at 4 months in the AETHERA study, with continued benefit seen over the 5-year follow-up period [17, 65] supports the rationale for extended use of consolidation therapy; in the case of the AETHERA study for up to 1 year after ASCT, to minimize the risk of relapse.

There are few publications characterizing the utility of similar markers in patients with HL [92–94]. Using a highly sensitive next-generation sequencing method, Oki et al. demonstrated the feasibility of identifying lymphoma-specific immunoglobulin gene segments in the peripheral blood of patients with cHL [92]. The authors postulated that this new blood-based method could be used to monitor disease burden and provide prognostic information for patients with cHL. In an exploratory analysis of patients enrolled in AETHERA, Bachanova et al. found that serum levels of thymus and activation-regulated chemokine may also be useful in identifying HL patients at increased risk of disease progression following ASCT treatment [94]. Moreover, Camus et al. determined that recurrent exportin 1 gene mutations in tumor and cell-free circulating DNA can be used as a novel biomarker in patients with cHL using a highly sensitive digital PCR technique [93]. Non-invasive methods utilizing cell-free circulating DNA to identify somatic mutations represent a promising advance in the management of HL. Molecular monitoring using cell-free DNA is emerging as a highly sensitive screening tool that has potential for future screening in hematologic malignancies [95, 96]. More studies are needed to further elucidate these methods fully in patients with HL.

The impact of post-ASCT consolidation therapy on quality of life (QoL) needs to be carefully considered, as patients may have already experienced poor health-related QoL during HDC and ASCT [97, 98]. Peripheral neuropathy is a common AE with anti-microtubule-directed agents, and was the most frequently occurring AE with brentuximab vedotin in the AETHERA study [65]. Although 67% of patients in this study experienced peripheral neuropathy, of which the majority were sensory events, only 13% of patients experienced grade 3 events, and there were no grade 4 events. In a QoL analysis of the AETHERA study, there were no significant differences in mean European Quality of Life five dimensions scores between patients with and without peripheral neuropathy within the brentuximab vedotin arm at any time point [99].

Consensus recommendations from ASBMT, CIBMTR, and EBMT provide strong support for the use of brentuximab vedotin as consolidation/maintenance after ASCT in brentuximab vedotin-naïve patients with cHL based on data from the AETHERA study (grade A recommendation; there is good research-based evidence to support the recommendation) [36]. Authors did not provide any specific guidance on use of brentuximab vedotin salvage therapy and the expert panel also assigned a grade C recommendation (the recommendation is based on expert opinion and panel consensus) to the use of brentuximab vedotin as post-ASCT consolidation/maintenance in patients with limited prior exposure to brentuximab vedotin (4–6 cycles) [36]. With the approval of brentuximab vedotin (plus doxorubicin, vinblastine, and dacarbazine) in previously untreated patients with advanced cHL there will likely be future changes to the currently recommended post-transplant strategies.

## Conclusions

In summary, post-ASCT consolidation (including radiotherapy) improves outcomes in patients with R/R HL who require ASCT, with early intervention providing the greatest benefits. More potent frontline and pre-ASCT salvage regimens also show promise in improving outcomes in this patient group, however, they have not yet been formally tested in phase III studies. Checkpoint inhibitors may be used for patients who have already received brentuximab vedotin. Approval of novel targeted agents is expected to further improve outcomes and provide additional treatment options in the coming age of personalized medicine.

## Data Availability

Not applicable.

## Abbreviations

ABMT: Autologous bone marrow transplant
ABVD: Doxorubicin, bleomycin, vinblastine, and dacarbazine
ADC: Antibody-drug conjugate
AEs: Adverse events
aICE: Augmented ifosfamide, carboplatin, and etoposide
allo-SCT: Allogeneic SCT
ASBMT: American Society for Blood and Marrow Transplantation
ASCT: Autologous stem cell transplantation
ASHAP: Doxorubicin, methylprednisolone, high-dose cytarabine, and cisplatin
B + DHAP: Brentuximab vedotin + DHAP
BEACOPP: Bleomycin, etoposide, doxorubicin, cyclophosphamide, vincristine, procarbazine, and prednisone
BEAM: Carmustine, etoposide, cytarabine, and melphalan
BeGEV: Bendamustine, gemcitabine, and vinorelbine
BrESHAP: Brentuximab vedotin + ESHAP
CAR-T: Chimeric antigen receptor-modified T cell
CD30: Cluster of differentiation 30
cHL: Classical HL
CIBMTR: Center for International Blood and Marrow Transplant Research
CMR: Complete metabolic response
CR: Complete response
Dexa-BEAM: Dexamethasone plus BEAM
DHAP: Dexamethasone, high-dose cytarabine, and cisplatin
EBMT: European Group for Blood and Marrow Transplantation
EBV: Epstein-Barr virus
EFS: Event-free survival
EPOCH: Etoposide, prednisone, vincristine, cyclophosphamide, and doxorubicin
ESHAP: Etoposide, methylprednisolone, high-dose cytarabine, and cisplatin
ESMO: European Society for Medical Oncology
FFTF: Freedom from treatment failure
GDP: Gemcitabine, dexamethasone, and cisplatin
GELTAMO: Grupo Español de Linfomas y Trasplantes de Médula Ósea
GemOx: Gemcitabine and oxaliplatin
GHSG: German Hodgkin Study Group
GVD: Gemcitabine, vinorelbine, and pegylated liposomal doxorubicin
HDC: High-dose chemotherapy
HL: Hodgkin lymphoma
HR: Hazard ratio
ICE: Ifosfamide, carboplatin, and etoposide
IGEV: Ifosfamide, gemcitabine, vinorelbine, and prednisolone
IVE: Ifosfamide, etoposide, and epirubicin
IVOx: Ifosfamide, etoposide, and oxaliplatin
LMP1: Latent membrane protein 1
LYSA: Lymphoma Study Association
MCL: Mantle cell lymphoma
MINE: Mitoguazone, ifosfamide, vinorelbine, and etoposide
MRD: Minimal residual disease
NCCN: National Comprehensive Cancer Network
NHL: Non Hodgkin lymphoma
NR: Not reported
ORR: Objective response rate
OS: Overall survival
PCR: Polymerase chain reaction
PD-1: Programmed cell death-1
PET: Positron-emission tomography
PFS: Progression-free survival
PR: Partial remission
pts: Patients
Q3W: Every 3 weeks
QoL: Quality of life
R/R: Relapsed/refractory
SCT: Stem cell transplantation
SD: Stable disease
SFGM: Société Francaise de Greffe de Moelle
TTSST: Time to second subsequent therapy
